# Monkeys share the neurophysiological basis for encoding sound periodicities captured by the frequency-following response with humans

**DOI:** 10.1038/s41598-017-16774-8

**Published:** 2017-11-30

**Authors:** Yaneri A. Ayala, Alexandre Lehmann, Hugo Merchant

**Affiliations:** 1 Instituto de Neurobiología, UNAM, Campus Juriquilla, Boulevard Juriquilla No. 3001, Querétaro, Qro. 76230 Mexico; 2 Department of Otolaryngology Head & Neck Surgery, McGill University, Montreal, QC Canada; 3 International Laboratory for Brain, Music and Sound Research (BRAMS), Center for Research on Brain, Language and Music (CRBLM), Pavillon 1420, Montreal, QC H3C 3J7 Canada; 4 Department of Psychology, University of Montreal, Montreal, QC Canada

## Abstract

The extraction and encoding of acoustical temporal regularities are fundamental for human cognitive auditory abilities such as speech or beat entrainment. Because the comparison of the neural sensitivity to temporal regularities between human and animals is fundamental to relate non-invasive measures of auditory processing to their neuronal basis, here we compared the neural representation of auditory periodicities between human and non-human primates by measuring scalp-recorded frequency-following response (FFR). We found that rhesus monkeys can resolve the spectrotemporal structure of periodic stimuli to a similar extent as humans by exhibiting a homologous FFR potential to the speech syllable /da/. The FFR in both species is robust and phase-locked to the fundamental frequency of the sound, reflecting an effective neural processing of the fast-periodic information of subsyllabic cues. Our results thus reveal a conserved neural ability to track acoustical regularities within the primate order. These findings open the possibility to study the neurophysiology of complex sound temporal processing in the macaque subcortical and cortical areas, as well as the associated experience-dependent plasticity across the auditory pathway in behaving monkeys.

## Introduction

The extraction and encoding of the time-varying features of acoustic signals is fundamental for complex skills. For example, successful vocal communication requires the dynamic sampling and neural representation of the spectral and temporal information of vocal sounds at multiple time scales^1^. The fidelity of neural encoding of acoustic regularities can be captured by the frequency-following response (FFR), an event-related potential reflecting synchronous phase-locked activity of different subcortical and cortical brain areas to the fundamental frequency (F0) and its harmonics of the sound^2–5^. The FFR mimics the envelope and/or fine structure of a sound waveform, capturing most of the temporal features of the evoking sound with extreme granularity, including the duration and periodicity of a stimulus.

The FFR has become increasingly popular as a neural biomarker of the stability of sound processing that correlates with literacy^6–12^ and beat entrainment capabilities^13–15^, revealing an intricate coupling between the auditory and motor systems^16,17^. More recently, it has been shown that the FFR can also act as a diagnostic tool for mild traumatic brain injuries^18,19^. Importantly, the FFR exhibits flexibility at different time scales and can be modified by changes in the stimulus dynamics^20–22^ and by short^23–25^ and long-term experiences^26,27^. Short-term interventions, such as auditory or linguistic training, enhance the neural encoding of the F0 which accompanies improvements in perceptual skills^25,28^. Thus, the FFR can act as an index of experience-dependent plasticity of sensory processing.

Because of the clinical and empirical relevance of the FFR, it is of timely interest to disentangle the neurophysiological underpinnings behind the extraction of temporal regularities of complex sounds, its experience-dependent plasticity as well as its association with sensorimotor capabilities. To do so, a mandatory step is to determine the commonalities in the neuronal sensitivity to temporal periodicities across humans and animals. Non-human primate is the closest animal model of human brain structure and function and they are able to discriminate speech sounds^29,30^, as well as to detect^31,32^ and entrain movements to isochronous auditory metronomes^33–41^. Hence, we compared the scalp-recorded response to the /da/ periodic syllable between human and rhesus monkeys. We hypothesized that the neural evoked responses in rhesus monkeys will show similar properties to the humans′ FFR, since the anatomy and physiology of the early and intermediate stages of the auditory pathway are conserved between the *Homo sapiens* and the macaque^42,43^.

## Results

Scalp-recorded FFR potentials were obtained from 18 human subjects and two monkeys who passively listened to a /da/ syllable of 40 ms duration^2^. Alternating polarity stimuli were delivered using a previously validated free-field method^44^. We used the same rate (11.1 Hz) and intensity (85 dB SPL) for both species. Temporal and spectral features of the added average of the electroencephalographic (EEG) response to stimulus of positive and negative polarity were estimated for each subject.

### Comparable FFR in human and monkey

**Figure 1:**
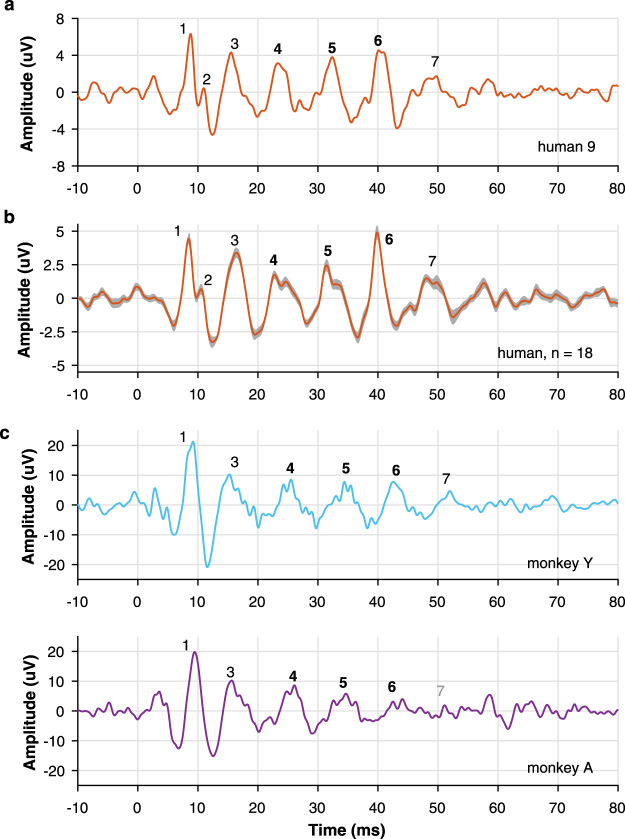
Human and macaque FFR to speech syllable /da/ in the time domain. (**a**) FFR of a representative human case. (**b**) Grand average FFR of the human population is shown as the mean ± SEM (shaded area). (**c**) FFR in two macaques, Y and A. Onset and sustained response peaks are numbered from 1–7. Peaks corresponding to the sustained part of the FFR are in bold. Note the ‘not reliable’ peak in gray in monkey A.

We found that the monkey FFR to the extensively studied /da/ syllable exhibits a comparable morphology to that of humans. The auditory evoked potential recorded in humans reliably replicated the FFR potential described with close^2,45–47^ and free-field protocols^44^. The human FFR exhibited seven characteristic positive peaks comprising the typical transient (peaks 1–3, 7) and sustained features (peaks 4–6) of the response (Fig. 1a,b). Using the same recording set-up and stimulation protocol, we observed that the monkey FFR exhibited six out of the seven peaks of the human response, including the three sustained elements that reflect the encoding of stimulus periodicity (Fig. 1c, Table 1). Peak 2 corresponding to stopping the consonant was absent in both monkeys, as well as the offset peak 7 in monkey A. The other transient peaks corresponding to the sound onset (peak 1) and to the transition between stop burst and onset of voicing (peak 3) were clearly identified. Importantly, analogous periodic peaks comprising the steady-state FFR segment were identified and labeled as 4–6 in both species. We performed a bootstrapping method with a 95% confidence interval (CI) to compare the FFR components in both species. The amplitude and latency of most peaks were larger in monkeys than in humans (Table 2). An accurate encoding of the F0 of the sound was revealed by the inter-peak intervals of the sustained FFR components. The 4–5 and 5–6 interpeak intervals showed a similar duration (~8.6 ms) to the period of the F0 of the stimulus (103–121 Hz) in the two primate species. Specifically, in humans, the 4–5 and 5–6 interpeak intervals were of 8.4 (interquartile range, IQR: 1.62) and 8.42 ms (IQR: 0.68), respectively. Similar values were obtained in monkeys at the 4–5 (monkey Y: 10.16, monkey A: 8.6 ms) and 5–6 interpeak intervals (monkey Y: 8.11, monkey A: 9.42 ms).

**Table 1:** Amplitude and latency of response peaks estimated from the added-average response.

	**Peak 1**	**Peak 2**	**Peak 3**	**Peak 4**	**Peak 5**	**Peak 6**	**Peak 7**
**Amplitude (uV)**							
Human	4.80 (1.22)	0.33 (1.85)	3.81 (1.44)	2.63 (1.69)	3.19 (0.70)	4.76 (2.11)	2.91 (1.38)
Monkey Y	19.34	n.r.	9.12	5.66	6.11	7.24	4.02
Monkey A	18.49	n.r.	9.62	7.53	5.13	3.22	n.r.
**Latency (ms)**							
Human	8.37 (0.55)	10.58 (0.37)	16.15 (1.27)	23.28 (1.27)	31.55 (1.35)	39.91 (0.45)	48.51 (1.8)
Monkey Y	9.19	n.r.	15.25	24.26	34.42	42.53	51.99
Monkey A	9.47	n.r.	15.62	26.06	34.66	44.08	n.r.

Human data (n = 18) is shown as the median and the 25^th^ and 75^th^ IQR are in parenthesis. n.r., not reliable.

**Table 2:** Average amplitude and latency across epochs of response peaks for the human and monkey groups.

	**Peak 1**	**Peak 3**	**Peak 4**	**Peak 5**	**Peak 6**
**Amplitude (uV)**					
Human	4.63 (4.24–5.02) n = 18 × 6400	4.12 (3.75–4.53) n = 18 × 6400	2.68 (2.30–3.06) n = 18 × 6400	3.33 (2.94–3.72) n = 18 × 6400	4.94 (4.54–5.33) n = 18 × 6400
Monkey	18.91 (17.36–20.41) n = 2 × 6400	9.36 (7.87–10.85) n = 2 × 6400	6.59 (5.13–8.08) n = 2 × 6400	5.62 (4.17–7.09) n = 2 × 6400	5.23 (3.71–6.72) n = 2 × 6400
**Latency (ms)**					
Human	8.38 (8.38–8.39)	15.99 (15.98–15.99)	23.42 (23.41–23.42)	31.84 (31.83–31.85)	39.86 (39.86–39.87)
Monkey	9.32 (9.31–9.33)	15.44 (15.43–15.45)	25.28 (25.26–25.29)	34.69 (34.68–34.70)	43.31 (43.29–43.32)

Data is shown as the mean and bootstrap 95% CI in parenthesis. The total number of epochs (n=) is indicated as subject *x* epochs.

### Stimulus-response temporal correlation

**Figure 2:**
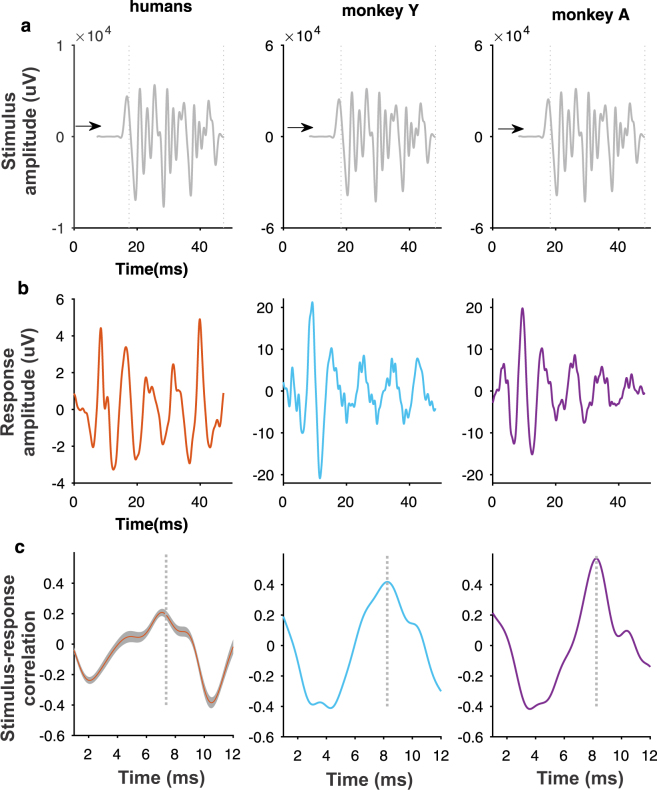
Stimulus-response correlation. (**a**) Low-pass filtered version of the stimulus /da/ syllable. Stimulus onset is shifted in time (arrow) to account for the time lag resulting in maximal correlation between signals with the aim of maximizing the visual coherence between stimulus and response waveforms. The dotted lines delimitate the sustained part of the stimulus (10–40 ms). (**b**) FFR waveform of human subjects (grand average) and macaques (individual averages). (**c**) Correlation coefficients between stimulus and response as a function of the time shift between them. The maximal correlation is reached at time displacement of 7–8 ms indicated by the dotted line.

Cross-correlation analysis demonstrated that the monkey FFR reliably captures the morphology and timing of the stimulus waveform. The estimation of the stimulus-response correlation constitutes a validated measure of similarity between signals^2^. Likewise, the time shift between both signals resulting in maximum correlation is used as an objective estimation of the response onset^2,7,45^. Stimulus and response were maximally positively correlated at time lags of 7.23 (IQR: 0.99) and 8.25 ms in human and monkey subjects, respectively (Fig. 2). These time lags match the neural transmission delay previously reported in human studies^2,45^ and the latency of the first peak observed in human and monkey response (Table 1). A significant stimulus-response correlation was obtained in human subjects (r = 0.3; IQR: 0.12, p < 4.17 e-5) and monkeys (monkey Y: r = 0.42, p = 2.37e-32; monkey A: r = 0.57, p = 7e-64).

### Spectral content of the FFR

**Figure 3:**
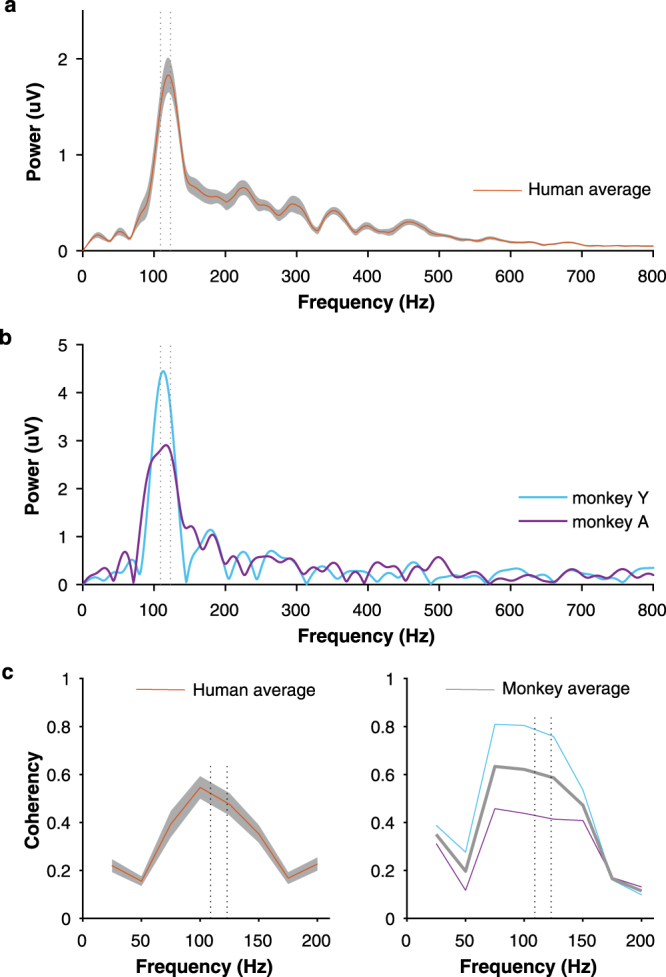
Spectral amplitude of the FFR. (**a**) Average frequency spectra of the FFR from the human population. (**b**) Frequency spectra of the individual FFR of each macaque. (**c**) Coherency index between the FFR segment and /da/ stimulus for human subjects (left) and monkeys (right). The mean ± SEM of frequency spectrum and coherency are shown for human data. The gray line shows the coherency average of the two macaque values. Dotted lines illustrate the F0 of the stimulus (103–121 Hz).

The evoked responses of monkeys also reflected the spectral features of speech sound by showing phase-locking properties to the F0 as observed in humans. The spectral information conveyed by the FFR segment (20–60 ms) was determined by the fast Fourier transform, and its association with the spectra of the stimulus was determined with the coherency analysis. Notably, the response amplitude (Fig. 3a,b) and coherency (Fig. 3c) peaked at the F0 of the stimulus, which ramps between 103 and 121 Hz. Also, the power of the response was averaged at the frequency ranges of 90–140 and 200–750 Hz, which include the F0 and harmonics of the stimulus, respectively^44^. The mean amplitude of the grand-added average at the F0 frequency range for human subjects (1.28 µV; IQR: 0.55) and monkey (monkey Y: 3.08 µV, monkey A: 2.48 µV) was larger than 1 µV, whereas the amplitude of the response at harmonic range was considerably small in both species (humans: 0.23 µV; IQR: 0.09, monkey Y: 0.26 µV, monkey A: 0.28 µV). Between groups, the average power across epochs of monkeys was larger than that of humans at the F0 and higher harmonics (HH) range (Bootstrapping, 95% CI). The average amplitude across epochs of humans was of 24.81 (CIs.: 24.71, 24.90) and 9.56 µV (CIs.: 9.54, 9.59) for the F0 and HH range, respectively. For the monkey group, the F0 and HH average amplitude was of 34.35 (CIs.: 33.96, 34.71) and 14.35 µV (CIs.: 14.21, 14.46), respectively.

### Intrinsic properties of the FFR

**Figure 4:**
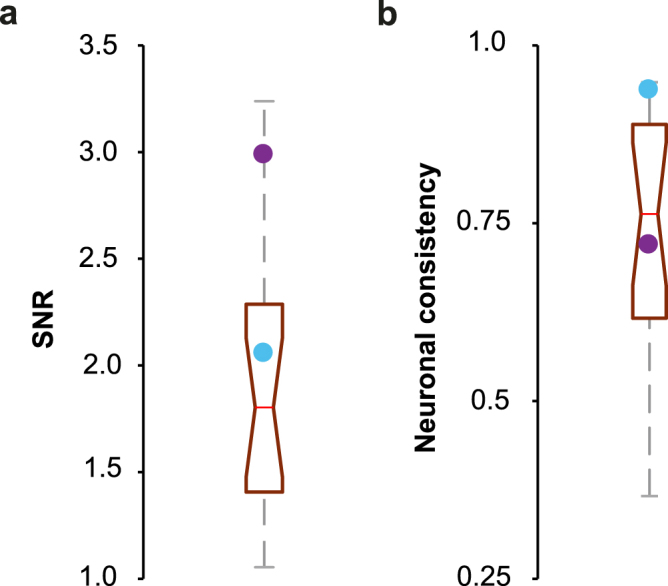
Intrinsic measures of the FFR. (**a**) Signal-to-noise ratio (SNR) between the FFR segment (20–60 ms) and baseline period (−10 ms) estimated by root-mean-square values. Data is shown as a box plot for the human data and as discrete values for monkeys (blue: monkey Y; purple, monkey A). The red line within each box represents the median values, the edges of the box delimit the 25^th^ and 75^th^ percentiles and the whiskers indicate the 10^th^ and 90^th^ percentiles. (**b**) Neural consistency index estimated by correlating the response waveforms resulting from averaging the odd and even epochs.

In both species, a robust neural representation of sound periodicities over time was captured by the FFR. The magnitude of neural activation was estimated by the signal-to-noise ratio (SNR), and neural consistency was calculated by the correlation between odd and even subaverages as previously described^44,48^. The amplitude of monkey (monkey Y: 2.05, monkey A: 2.98) and human (1.80; IQR: 0.88) FFR was above noise level (Fig. 4a). The average SNR across epochs indicated a slightly larger neural activation in monkey response (1.66, CIs.: 1.50, 1.87) than in human response (1.19, CIs.: 1.18, 1.19). The FFR was largely consistent across stimulus presentations in both human subjects (r = 0.76; 0.62, 0.89, p < 2.06e-32) and monkeys (monkey Y: r = 0.94, p = 0; monkey A: 0.72, p = 1.82e-15; Fig. 4b).

## Discussion

We found that rhesus monkeys exhibit a homologous human FFR, revealing a shared neural sensitivity to acoustic periodicities. Like humans, monkeys are capable of resolving the spectrotemporal structure of the synthesized speech syllable /da/ (Figs 1 and 3). Monkey FFR exhibits the same number of sustained phase-locked peaks consistently described in the human FFR^2–4^ and occurring at similar latencies (Tables 1 and 2). Furthermore, a high-fidelity and stable representation of the temporal structure of sound occurs in the FFR of both species (Figs 2 and 4).

The periodic activity of the monkey FFR reproduces the F0 of the stimulus as observed in human responses, indicating comparable phase-locking abilities to fast temporal information such as subsyllabic cues in both species. Imaging studies in humans using the diphthong /da/ stimulus have demonstrated that auditory areas, including the cochlear nucleus, inferior colliculus, medial geniculate body and auditory cortex, contribute to the scalp-recorded FFR potential^49,50^. Furthermore, it has been shown that the lemniscal auditory pathway comprising the central nucleus of the inferior colliculus, ventral division of the medial geniculate body together with the core region of the auditory cortex has a strong contribution to the FFR^17^. Lemniscal auditory neurons are highly sensitive to the physical features of the sound exhibiting fast and high-fidelity responses which contrast with the habituating responses to unvarying stimuli of the non-lemniscal neurons^51–53^. Whether analogous auditory neural structures encode temporal periodicities in the order of milliseconds in both species remains to be addressed. Regardless, the principles of organization of auditory ascending pathway in which specialized neurons encoding the acoustical properties are tightly interconnected seems to be a general feature across mammals^51,52,54^.

In the rhesus monkey, two ascending auditory pathways are identified: the ‘direct’ and ‘indirect’ pathways, both originating in the cochlear nucleus subdivisions and projecting directly or passing though the superior olivary complex to the inferior colliculus respectively^55–57^. Although there are no studies on the response of brainstem and midbrain neurons to periodic sounds in monkey, it has been shown that neurons of the cochlear nucleus^58^ and central nucleus of the inferior colliculus^59^ of guinea pigs exhibit a similar pattern of periodic activity as synthetic speech sounds. In rhesus monkey, cortical phase-locked activity to periodic speech sounds including vowels^60–62^ and the /da/ syllable^63^ has been recorded in the primary auditory cortex. Those studies showed that cortical neurons can resolve the spectral fine-structure (harmonics) as well as the envelope of speech sounds using a ‘rate-place’ code^60–63^. Furthermore, the lag between the periodic peaks of response and /da/ syllable was of 8.5 ms^63^, similar to the lag observed in the scalp-recorded FFR (Fig. 2). Overall, animal studies indicate that neurons along the mammalian auditory ascending pathway exhibit a phase-locked patterning of activity that follows the acoustic periodicities in the order of milliseconds.

**Figure 5:**
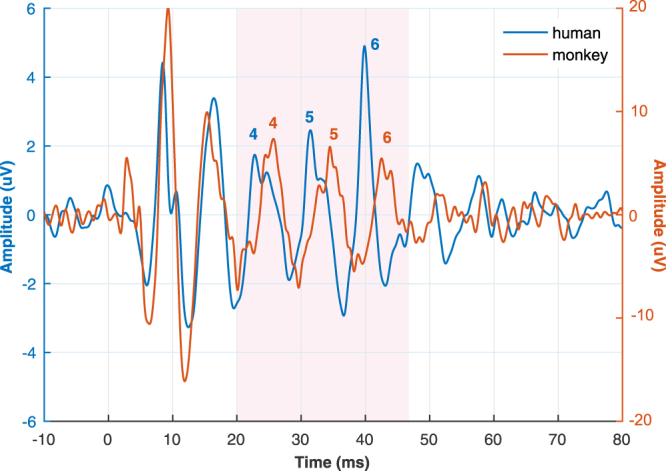
Human and monkey FFR potential. Average FFR across human subjects (n = 18) and monkeys (n = 2) recorded in the head vertex. The overall morphology of both primate responses exhibited three sustained peaks labeled as 4–6 reflecting the phase-locked activity of large neural ensembles to the fundamental frequency of the /da/ stimulus. Note the absence of peak 2 and larger amplitude and latencies in monkey FFR.

Further intracerebral recordings are needed to determine the contribution of each of the auditory structures on the scalp-recorded monkey FFR, as well as its differences with the human FFR. Monkey FFR exhibits larger peak amplitude and latency (Fig. 5, Tables 1 and 2). Early^64^ and middle latency evoked responses^65^ in monkeys also have a larger amplitude than human responses, which could be partially explained by the smaller thickness of the monkey skull and larger ratio between sizes of electrode and neural generators. The longest latencies of monkey FFR (Fig. 5, Tables 1 and 2) could reflect slower dynamics of activation of auditory generators in response to acoustic periodicities. Although, a second possibility is that longer latencies resulted from larger and more frequent movement artifacts occurring in monkey recordings, since animals were not head-restrained and could move spontaneously. While peak 2 in human average response was small (Fig. 1, Table 1) and in some individual responses it was not observed, this peak was absent in both monkey FFRs. Peak 2 is a transient feature of human FFR associated with the transition between the broadband stop burst and onset of voicing associated with the /d/ and /a/ segment, respectively^44,47^. Thus, it is possible that humans have a larger sensitivity to burst-voice transition or that the analogous neural structures in monkeys elicit a weak response not captured by the scalp recording.

Importantly, a shared capacity to track isochronous regularities in the two primates is indicated by the interpeak intervals and spectra analysis of the sustained features of human and monkey FFR. Nevertheless, recent studies have shown a gradient in the sensitivity to represent the metrical structure of complex sounds across the primate order^35,66–70^. Rhesus monkeys are able to detect regular acoustical metronomes^31,32^ but not to perceive the pulse in rhythmic patterns with a complex meter as humans do^31^. Accordingly, the gradual audiomotor evolution hypothesis proposes that the meter hierarchy at which primate species can perceive and entrain movements may have developed as a consequence of a gradient of anatomofunctional changes in the auditory and motor systems^35^. Further comparative studies are needed to determine the stage at which the structure and function of circuits diverges to account for different meter hierarchy sensitivity.

Similarly, it is of timely interest to study how early encoding of acoustic regularities is exploited and modulated by downstream sensory and motor neural circuits to support perception and production of more complex temporal patterns^69–71^. Human studies suggest an overlap between auditory circuits underlying the FFR and higher level neural circuits involved in beat entrainment. It has been observed that subjects that can entrain, with high precision, to an external beat have a larger phase locking and inter-trial consistency of the FFR potential^12,72^. These observations strongly suggest that the precise representation of the temporal structure of sound occurring at low hierarchical levels are used to plan and align motor outputs in phase to the stimulus^17,70,73,74^. Furthermore, top-down signals also sharpen the early processing of temporal regularities, as revealed by the finding that FFR strength is modified by short-term training protocols^23,75^ and on-line cognitive factors^46,76^.

Non-human primates are able to perceive and respond predictively to isochronous auditory sequences^33,36,37^ and notably, the stimulus-response correlation and the neural consistency of monkey FFR was comparable (Fig. 4b) to human indices, reflecting a stable and faithful representation of the regular temporal structure of sound over time in both species. Therefore, the neurophysiological basis of audio-motor entrainment^34,36,37,77^ and the associated experience-dependent plasticity of the FFR potential can be studied in the behaving monkey to understand the mechanisms behind the correlation between the extraction of periodic auditory features and precision of synchronization performance observed in humans.

In summary, our findings reveal a conserved neural tracking accuracy for stimulus regularities between human and non-human primates. Thus, the rhesus monkey can be a suitable animal model not only to investigate the neurophysiological basis of the FFR, but also to study the neural mechanisms behind the association between beat entrainment abilities and experience-dependent auditory plasticity.

## Methods

### Subjects

Non-invasive electroencephalographic activity was recorded on two male rhesus monkeys (*Macaca mulatta*, 10 and 9 years old, 6–7 kg BW) and 18 human subjects (mean age ± SD: 26 ± 4 years, 10 females) while passively listening to free-field auditory stimuli as previously demonstrated to elicit reliable FFR responses^44^. Subjects had no history of hearing disorders and all gave written informed consent. Experimental procedures on human subjects were approved by the UNAM Research Ethics Committee and were compliant with the Declaration of Helsinki. All the animal care, housing, and experimental procedures were approved by the National University of Mexico Institutional Animal Care and Use Committee and strictly conformed to the principles outlined in the Guide for Care and Use of Laboratory Animals (NIH, publication number 85–23, revised 1985). The researchers and animal care staff monitored the two monkeys daily to ensure their health and well-being. To ameliorate their condition of life, we routinely introduced toys (often containing food items that they liked) to their home cage (1.3 m^3^) environment to promote their exploratory behavior.

### Acoustic stimulus

The acoustic stimulus was the 40 ms consonant–vowel /da/ syllable, one of the most extensively used speech sounds in FFR human studies^2^. The stimulus has an F0 that rises linearly from 103 to 121 Hz; voicing begins at 5 ms and onset noise burst occurs during the first 10 ms. The acoustic stimulus was resampled at 24414 Hz and a cosine ramp of 2 ms was applied at the beginning and end of the stimulus (*GenerateEnvelope*, Psychoacoustics ToolBox, MathWorks, Natick, MA, USA)^47^.

### Stimulation procedure

Human subjects and monkeys were awake; head unrestrained and comfortably seated inside an electrically isolated and sound-attenuated room without performing any behavioral task. To prevent drowsiness and minimize motion, human subjects watched a silent subtitled movie from a laptop running on battery power. The /da/ syllable was binaurally delivered through magnetically shielded studio monitors (KRK 5-G3, USA) with a 55-cm distance between the speakers and the subject’s ears. Blocks of 2,000 stimuli in alternating positive and negative polarity at a rate of 11.1 Hz and an intensity of 85 dB SPL were delivered. Sound intensity was measured with an omnidirectional microphone (M 101, Beyer Dynamic, Germany) placed at the site of subject’s head. The /da/ stimulus blocks were randomly interspersed among blocks of other simple (click) and complex sounds (human word and macaque vocalizations) in two recording sessions per subject. Sound delivery was controlled through an RZ2 TDT BioAmp Processor (TDT, System 3) using customized RPvdsEx circuits. The audio file (.wav) containing the /da/ stimulus was loaded into RPvdsEx buffers and triggered at the desired rate and voltage.

### Recording set-up

Scalp-recorded activity was collected with Grass gold-cup electrodes (Natus Neurology, #FS-E5GH-60) at Fz, Cz, Pz, F3 and F4 sites according to the 10/20 EEG system. The reference and ground electrodes were placed on the inion and forehead, respectively. Electrodes were attached to the subject’s scalp using conductive EEG paste (Ten20, D.O. Waver and Company, USA). The scalp of the macaques was shaved and the scalp of human subjects was cleaned with mild abrasive gel (Nuprep, D.O. Weaver and Company, USA) before each recording session to reduce scalp impedance. Here, the signal recorded at the Cz site was analyzed since it exhibited the largest SNR and it has been reported in previous EEG monkey studies^31^. Furthermore, artifacts due to the postauricular muscle reflex are diminished by using the vertex-inon derivation^78^.

### Signal acquisition

The EEG signal, as well as the sound trigger and waveform, were simultaneously acquired at a sampling rate of 24414.0625 Hz using the same RZ2 TDT BioAmp Processor (TDT System 3) that controlled the presentation of stimuli. The signal was re-referenced to the signal recorded at inion and amplified using the Medusa preamplifier (RA16PA, TDT systems). Next, the EEG signal was band-pass filtered between 1–3000 Hz, and a notch filter at 60 Hz was applied to the signal to remove the line frequency using RPvdsEx TDT filter components. The signal timeline was corrected by 1.75 ms to compensate for the delays introduced by the sound delivery set-up.

### Signal processing

A total of 8,000 stimulus presentations were recorded for macaques and human subjects. Half the set of epochs corresponded to stimuli of positive polarity and the other half to stimuli of negative polarity. Twenty percent of epochs having the largest amplitude were discarded from each individual set of epochs regardless of the stimulus polarity to remove muscle and movement artifacts that ensure a stable SNR^50^. Next, the signal was further band-pass filtered (100–1000 Hz, Butterworth 4^th^-order filter) and epoched with respect to stimulus onset (−10–80 ms). EEG epochs were sorted into positive and negative polarity stimulus presentations and sub-averages were computed for each polarity condition. The added polarity grand average was obtained and used for further analysis to avoid any transduction stimulus artifact and to minimize the cochlear and microphonic potentials^2^. The median followed by the 25–75^th^ IQR are indicated for human data while individual values are reported for monkeys.

### Signal analysis

FFR analysis was conducted using custom routines in Matlab 2016b (The MathWorks Inc, Natick, Massachusetts, US) and built functions from the Brainstem Toolbox (BT_2013) and Chronux Software (Chronux 2.12). The EEG signal was analyzed following measures of acoustic and intrinsic properties, as well as of response reliability typically employed in FFR studies in open-^6^ and close-field stimulation experiments^45^.

## Acoustic properties

### Peak amplitude and latency

Prominent, on-going peaks were identified following the nomenclature used by Lehmann and colleagues in a previous FFR study in humans using open-field stimulation^44^. For human responses, we used the latency of the peaks at the grand average to guide the peak identification in individual responses. A peak was coded ‘reliable’ and included in the analysis if visual observation by two raters revealed that the peak’s amplitude was above the pre-stimulus amplitude. The amplitude of each peak was estimated as the mean value from a window of ±0.5 ms around the maximum value. The latency was estimated as the time relative to the onset of the stimulus corresponding to the maximum amplitude. The response window containing the FFR segment was set at 20 to 60 ms in both the human and monkey response.

### Temporal correlation between stimulus and response waveform

The FFR segment was correlated with the 10–40 ms portion of a low-pass filtered version of the stimulus (500 Hz, Butterworth 3^rd^-order filter) that includes its harmonic segment^7^. The low-pass filtered version of the stimulus was employed to accentuate the envelope of the stimulus^7^. The function *bt_xcorrelation* of the BT_2012 Toolbox was used to calculate Pearson's correlation coefficients.

### Spectral amplitude

Response amplitudes in the spectral domain were computed for the corresponding FFR portion using the *bt fftsc* function from the BT_2013 toolbox^2^. A 2 ms on– 2 ms off Hanning ramp was applied to the waveform. Zero padding was employed to increase the number of frequency points where spectral estimates were obtained. Average spectral amplitude was calculated for two different frequency ranges around the fundamental frequency (F0: 90–140 Hz) and its harmonics (H: 200–750 Hz)^44^. To estimate the strength of association between the spectral content of the stimulus (0–40 ms) and the response at F0, a coherency index was estimated using the *coherencyc* function of Chronux Toolbox^79^. Coherency is the cross-spectrum magnitude of stimulus and response signals at frequency index *j*, divided by the power spectrum magnitude of each signal at frequency index *j*. For this aim, response subaverages of 800 epochs randomly selected with replacement were calculated for each subject.

## Intrinsic properties

### Root mean square and signal-to-noise ratio

To estimate the response strength, we estimated the magnitude of neural activation in the FFR and 10 ms pre-stimulus period using the root mean squared values (RMS). Subsequently, the SNR was estimated as the ratio between the RMS of the FFR segment and the pre-stimulus segment^2^. The RMS and SNR were estimated using the *bt_rms* function of BT_2013 toolbox.

### Neural consistency

Neural consistency was estimated for individual FFRs to assess the extent to which the brainstem's representation of sound varies from trial to trial. The FFR portions of the neural signal from each subject were split into even-odd halves taking into consideration both stimulus polarities^44^. In detail, the epochs were first sorted into positive- or negative-polarity trials, then, the epochs of each set of polarity were further split into odd or even trials according to their serial order within each set. Finally, even-ordered positive polarities were averaged, even-ordered negative polarities were averaged and those two were further averaged into an even-ordered FFR sub-average. The same procedure was done for odd-ordered epochs. Finally, the consistency index was computed as the correlation between the even-ordered FFR sub-average and odd-ordered FFR sub-average, with coefficient values close to one representing a more consistent response^44,48^.

### Comparison between human and monkey data

Human and monkey groups were compared using the bootstrapping method on trial-by-trial estimations of (1) response peak amplitude and latency, (2) power spectrum and (3) SNR of the FFR segment. This method relies on random sampling with replacement to determine the confidence intervals of sample estimates^80^. A total of 10,000 bootstrap samples were obtained. Non-overlapping 95% CIs between groups indicated significant difference. For the power spectrum and SNR analysis, the human group comprised 18 subjects ×6,400 epochs, while for the response peak analysis, the number of subjects varied according to the identification (reliable or not reliable) of each peak (indicated in Table 2). The monkey group comprised two subjects ×6,400 epochs.
